# Impact of Internal and External Factors on the Profitability and Financial Strength of Insurance Groups

**DOI:** 10.1007/s11294-023-09873-y

**Published:** 2023-05-31

**Authors:** Evaggelia Siopi, Thomas Poufinas

**Affiliations:** Department of Economics, Democritus University of Thrace, Komotini, Greece

**Keywords:** Insurance groups, Profitability, Financial strength, European sovereign debt crisis, Solvency II, G22, L25

## Abstract

This paper studies the impact of internal and external factors on the profitability and financial strength of insurance groups across the European Union, focusing on the effect of the European sovereign debt crisis and the Solvency II Directive, which has not been addressed by the literature. The findings reveal that the efficiency of accounts receivable management and the state of the economy have a significant positive impact, whereas the underwriting risk and size have a significant negative impact on profitability and financial strength. Furthermore, long-term interest rates exert a significant positive influence, whereas the purchase of reinsurance, size of the domestic market, market structure, and inflation exert a significant negative influence on profitability. The European sovereign debt crisis has a significant negative impact on profitability and a positive but insignificant impact on financial strength. Solvency II has no statistically significant impact either on profitability or on financial strength. This paper contributes to the literature through the identification of the impact of the European sovereign debt crisis and Solvency II, which has not been depicted so far, as well as the impact of a series of internal and external factors on profitability and, for the first time, on financial strength.

**Supplementary Information:**

The online version contains supplementary material available at 10.1007/s11294-023-09873-y.

## Introduction

The global financial crisis, followed by the sovereign crisis, hurt the economies of all countries and affected primarily the banking sector. In contrast, the solvency of the insurance sector as a whole did not appear to be threatened. Insurance companies were affected less than banks due to lower liquidity risk (Liedtke & Schanz, 2010) and limited systemic risk (Harrington, 2009) through their investment portfolios and concentrated exposures to credit and market risks, as well as financial guarantees. Such (contained) risks concerned both sides of the insurance company balance sheets (Schich, 2010).

The European insurance market faced significant and still-evolving changes in the supervisory and regulatory environment since January 1^st^, 2016, when the Solvency II Directive was first enforced. Its main objective was to ensure the protection of policyholder interests and to level the playing field in the insurance industry. The Solvency II Directive, the preparation of which started well in advance of its implementation, provided motivation for better managing the insurers’ risk profiles and enhancing risk management practices. Consequently, it can yield advantages for policyholders in terms of their protection, insurers in terms of capital savings, and all market participants in terms of a well-functioning system.

The purpose of this study is to unveil the first effects of Solvency II (if any) on the profitability and financial/ capital strength of European insurance groups over a period that includes the European sovereign debt crisis (ESDC) and the coronavirus (COVID-19) pandemic. Therefore, it contributes to the literature in three ways, providing evidence on the impact of the ESDC, impact of the implementation of the Solvency II regulatory framework, and determinants of profitability and financial strength of the European Union (EU) insurance sector. To achieve this, it develops a series of models that embed a set of internal (micro) and external (macro) factors applied to 23 EU-based insurance groups, over the period 2007-2021.

## Literature Review

In this paper’s investigation of the impact of the European sovereign debt crisis (ESDC), the introduction of the Solvency II Directive, as well as of other determinants on the profitability and financial strength of EU insurers, three relevant literature strands were considered. The first strand studied the effects of the global financial crisis (GFC) and the subsequent ESDC and was found to be rather limited. Initially noted was that these effects were felt by the regional economies with a certain delay, compared to when they were felt by the developed western economies (International Monetary Fund, 2008). The financial crisis and the subsequent recession imposed substantial changes on the institutional and business landscape in which the insurance industry operates. This crisis resulted from the complex interaction of market failures, global financial and monetary imbalances, inappropriate regulation, weak supervision and poor macro-prudential oversight (De Larosière et al., 2009). Clearly shown was that neither government regulation nor rating agencies anticipated the potential problems in a timely manner, which implied the need for a change in order to prevent problems recurring in the future. The impact of the financial crisis on the insurance industry demonstrated the complexity of insurers' exposures to risks and the multiplying influence on their overall solvency position, but it was not the same for all insurance companies. The crisis had the strongest negative impact on those insurers who were engaged with innovative financial products, were specialised and thus not well diversified, and had aggressive investment policies that produced investment portfolios with a considerable share of riskier assets, atypical of the insurance industry's traditional assets (Marović et al., 2010). Baluch et al. (2011) studied the impact of the crisis on European (among global and United States (U.S.)) insurers, finding that European insurers outperformed their U.S. counterparts. However, they also faced turbulent times when the daily losses of the respective indices were more than 5%. Looking at their stock prices, (non-United Kingdom (UK)) European insurers posted among the worst performances during the financial crisis. This might be explained by the positions they had in toxic assets and liabilities underwritten on these assets, the cross-holdings between banks and insurance companies (not as common in the UK) which created problems for insurers from their associated banking activities, the prevalence of a few big insurers with cross-bank ownership, and the use of softer forms of capital by certain insurers. The aforementioned discussion demonstrates that the literature has not sufficiently covered the impact of the crisis on European insurers’ profitability and has not addressed at all its impact on financial strength. This paper contributes to this literature strand by revisiting the question of whether the crisis affected profitability and addresses for the first time the effect on the financial strength of EU insurers.

The second literature strand studied the impact of the regulatory frameworks in which insurers operate, and is prior to the Solvency II regime. Dell’Ariccia and Marquez (2006) suggested that greater regulatory competition was positively and significantly related to various measures of insurer profitability. In anticipation of the implementation of Solvency II, Jurkonytė & Girdzijauskas (2010) noted that the Solvency II requirements were expected to have a negative impact in the short term on the financial performance of insurance companies, as evidenced by their findings on the Lithuanian non-life insurance companies. Gaganis et al. (2015) explored the effect of various regulatory policies on the performance of European insurers (life and non-life). Their findings indicated an inverted U-shaped relationship between return on assets (ROA) and regulations pertaining to capital adequacy, accounting and auditing requirements, and disclosures to the supervisors. Thus, it is evident that the literature has not addressed the impact of Solvency II on either profitability or financial strength. This paper attempts to cover this gap by analyzing the effects of Solvency II on the profitability and financial strength of EU insurers.

Finally, the third literature strand studied the determinants of the financial performance of insurance companies in certain European countries. Non-European countries were omitted though they demonstrate similar findings. Only one paper that studied the drivers of profitability in the EU at a country, but not firm, level, and a second article that did the same in four Central and Eastern European countries was identified. The remaining papers focused on one country. More specifically, Dorofti & Jakubik (2015) studied the external factors that affect the profitability of EU insurers (measured by ROA and return on equity (ROE)) using a cross-country EU aggregate dataset for the period 2005-2012. They realized that low interest rates, mediocre economic growth, inferior equity market returns and high inflation rates posted a negative effect on insurance profitability. Kramaric et al. (2017) explored the drivers of ROA and ROE in Croatia, Slovenia, Hungary and Poland over the period 2010-2014. They found that age and gross domestic product (GDP) per capita growth had a significant positive effect on ROE and only age had a similar effect on ROA.

At a country level, Shiu (2004) found that liquidity, interest rates, and underwriting profits had a positive impact, whereas inflation exerted a significant negative impact on performance (measured by investment yield, percentage change in shareholders’ funds and return on shareholders’ funds) in the UK non-life market. Also, in the UK (compared with the U.S.), Batool & Sahi (2019) saw that size, liquidity, GDP, cost per impression (CPI) and West Texas Intermediate posted significant positive leverage. Asset turnover and interest rate exerted a significant negative impact on ROA and ROE. In the U.S., leverage, asset turnover and CPI swapped signs.

Pervan et al. (2012) found that the claims ratio had a significant negative effect, whereas age, market share and past performance had a significant positive effect on ROE in Bosnia and Herzegovina. Burca & Batrinca (2014) found that the financial leverage, size, gross written premium (GWP) growth, underwriting risk, the risk retention ratio and the solvency margin were the main (internal and external) factors affecting ROA in Romania. The first, third, and fourth variables posted a significant negative impact, whereas the remaining variables exerted a significant positive impact on ROA. Kočović et al. (2014) similarly concluded that the combined ratio, financial leverage and the retention rate had a significant negative effect, whereas GWP growth, return on investment and size posted a significant positive effect on ROA in Serbia. In the same country, Marjanović & Popović (2020) realized that the years of operation, capital adequacy, and market share posted a significant negative effect, whereas investment performance and the GDP growth rate exerted a significant positive impact on ROA. Also in Serbia, Vojinović et al. (2022) observed that size, GDP, population growth rate, political stability and degree of specialization (in some models) were significantly positively correlated, whereas risk taking and inflation (in some models) were significantly negatively correlated with ROA and return on total premium.

Kripa & Ajasllari (2016) realized that growth rate, liabilities, liquidity and fixed assets were the key drivers of ROA in Albania. The growth rate exerted a positive effect, whereas the other three exerted a negative effect. Ortyński (2016) found that the underwriting activity and net operating expenses had significant negative impact, whereas size had a positive impact on profitability (measured by the profitability ratio of technical activity, ROA, ROE, the sales profitability ratio, profitability of subscribed capital and profitability of GWP) in Poland. The GDP growth rate and the motor GWP ratio exhibited a significant positive effect on the profitability ratio of technical activity.

Kosumi & Poposka (2018) studied the drivers of ROA in Kosovo to see that size exerted a positive effect, whereas liquidity and leverage exerted a significant negative impact on ROA. In the same country, Ahmeti & Iseni (2022) found that leverage, size and age had a significant impact on ROA. Leverage had a negative effect. The other two factors had a positive effect. Size and firm growth had a significant positive impact on net profit margin.

The literature has not investigated the internal (concurrent with external) factors of insurance profitability at an EU level (except for Dorofti & Jakubik (2015) for external factors). Further, there seems to be no research on the determinants of financial strength. This study addresses this gap as it attempts to identify both the internal and external factors of profitability and financial strength at a firm level for EU insurers.

The aforementioned discussion shows that the existing literature has not considered the influence of the ESDC and Solvency II on the profitability of an insurer. In addition, it has not captured the determinants of its financial strength. This paper offers a novel approach to fill these gaps by studying the impact of Solvency II and the crisis on the profitability and financial strength (introducing the equity-to-assets ratio as its proxy) of EU insurers. It also captures the effect of the determinants already considered in the literature, by employing the latest available data as per 2021.

## Research Methodology

This study examines the possible impact of the European sovereign debt crisis^1^and implementation of the Solvency II Regulatory Framework^2^ on the profitability and financial strength of insurance groups.The *return on assets (ROA)* and *return on equity (ROE)* were used as proxies for profitability and the *equity-to-assets ratio* was employed as a proxy for financial strength. The impact of a series of internal (micro) and external (macro) variables on these proxies was studied for a set of 23 selected insurance groups that operated and had a country of origin in the EUn. The period under examination was from 2007 to 2021 (15 years). These insurance groups were chosen based on data availability from Datastream (Refinitiv, 2022) for the variables and period under investigation. More specifically, out of the 51 listed insurance groups and companies domiciled and active in the EU, 29 insurance groups were initially selected, as data were missing for the insurance companies. Six of the insurance groups were dropped from the dataset as there were no data for the sub-period 2007-2011. This led to a set of 23 insurance groups for which data were available for all variables and the entire period 2007-2021 (Online Supplemental Appendix Table 1).

### Variable definitions

As mentioned, profitability was measured with *ROA* and *ROE*^3^ (Adams & Buckle, 2003) and financial strength was captured by the *ratio of equity to assets* of an insurance company. Under the Solvency II Directive, equity was broadly defined as assets less liabilities. At the firm level, the variables used in the models were the *reinsurance ratio,*^4^ the *ratio of invested assets to total assets*, *net premium to equity*, and *company size*. At the country level, the control variables used in the models (to reflect the main macroeconomic conditions under which insurance groups operate) were *GDP growth*, the *inflation rate*, and *long-term interest rates*. In addition, three country-and-industry-specific variables were considered: the *size of the domestic market*, *cumulative market share held by the five largest insurers*, and the (insurance) *penetration* (ratio). Finally, two dummy variables were included in the model, *Crisis* and *Solvency II*, which captured the European sovereign debt crisis and the Solvency II Directive implementation, respectively (Table 1).

**Table 1:** Definition of the model variables

Variable	Description	Alternative verbiage	Level
ROA	Profits before taxes to total assets	Profitability metric	Firm
ROE	Profits before taxes to total equity (assets-liabilities)	Profitability metric	Firm
Equity/Assets	Total equity (assets-liabilities) to total assets	(Ratio of) equity to assets; total equity to total assets; financial strength; financial solvency; capital strength; capitalization ratio	Firm
Ceded prem /Gross prem	Ceded premium to reinsurance over the total gross written premium	Ceded premium/gross premium; ceded premium to gross premium; reinsurance ratio; use of reinsurance; purchase of reinsurance	Firm
Inv/Assets	Invested assets to total assets	Investments/total assets; (ratio of) invested assets to total assets; (efficiency of the) management of accounts receivable	Firm
Net prem/Equity	Net premium written to Equity	Net premium/equity; net premium to equity (ratio); underwriting ratio; underwriting risk; (insurance) leverage ratio	Firm
Ln-size	(Natural logarithm of) total assets	Company size; firm size; the size of the insurance group	Firm
Ln-Market size	(Natural logarithm of) total premiums in each country where every insurance group is domiciled	Size of the domestic market; market structure	Country
Market share of the top five	Cumulative market share held by the five largest insurers	Five-firm concentration ratio; market share held by the five largest insurers; concentration in (domestic) insurance market	Country
GDP annual growth	Annual GDP growth	GDP growth annual %, gross domestic product annual growth; annual growth of GDP; state of the economy	Country
Inflation rate	Annual inflation rate		Country
Long-term interest rates	Refers to government bonds maturing in ten years (yield to maturity)		Country
Penetration (gross prem to GDP)	Gross Written Premium to GDP	Gross (written) premium to GDP; (insurance) penetration (ratio)	Country
Crisis (period)	European sovereign debt crisis period (2009-2012)		1: 2009-2012; 0: elsewhere.
Solvency II (period)	Implementation of the Solvency II Directive		1: 2016-2021; 0: elsewhere.

Source: ROA, ROE, Equity/Assets, Ceded prem/Gross prem, Inv/Assets, Net prem/Equity, and Ln-size were retrieved from the Datastream database (Refinitiv, 2022). Ln-Market size, and Market share of the top five were taken from Insurance Europe (2022). Long-term interest rates were retrieved from the Federal Reserve Bank of St. Louis (2022). GDP growth and Inflation rate were sourced from the World Development Indicators (World Bank, 2022). and Penetration (gross prem to GDP) was taken from OECD (2022).

*ROA*, *ROE*, the *ratio of equity to assets*, the *reinsurance ratio*, *ratio of invested assets to total assets*, *net premium to equity*, and *company size* were retrieved from the Thomson Reuters Datastream database (Refinitiv, 2022). The *size of the domestic market* and *cumulative market share held by the five largest insurers* were taken from Insurance Europe (2022). L*ong-term interest rates* were retrieved from the Federal Reserve Bank of St. Louis (2022). The (annual) *GDP growth* and *inflation rate* were sourced from the World Development Indicators (World Bank, 2022). The (insurance) *penetration ratio* (gross premium to GDP) was taken from the Organization for Economic Co-operation and Development (OECD, 2022).

### Econometric approach

This research followed a balanced panel data approach. The general empirical model that examined the factors affecting *ROA, ROE* and *the equity-to-assets ratio* as dependent variables (*DV*) was:

1 $${DV}_{i,t}=a+\beta {Firm}_{i,t}+\gamma {Country}_{i,t}+{\delta}_1 Crisis+{\delta}_2 SolvencyII+{\varepsilon}_{i,t}.$$

*Firm* and *Country* represented the vectors of firm-specific and country-specific variables respectively. The other two variables were the dummy variables for *Crisis* and *Solvency II*. To estimate this model, a panel-corrected standard errors model (PCSE) was adopted following Bailey & Katz (2011), and Jönsson (2005). The expected impact of the factors studied in the available literature is in Table 2.

**Table 2:** Overview of profitability determinants’ impact in the literature

Variable	Positive Impact	Negative Impact	Expected Impact
Ceded prem /Gross prem		Burca & Batrinca (2014);	-
Inv/Assets			+
Net prem/Equity		Burca & Batrinca (2014)	-
Ln-size	Burca & Batrinca (2014); Kočović et al. (2014); Vojinović et al. (2022); Ortyński (2016); Kosumi & Poposka (2018); Ahmeti & Iseni (2022); Batool & Sahi (2019)		+
Ln-Market size			?
Market share of the top five			?
GDP annual growth	Dorofti & Jakubik (2015); Kramaric et al. (2017); Marjanović & Popović (2020)		+
Inflation rate		Shiu (2004); Dorofti & Jakubik (2015); Vojinović et al. (2022)	-
Long-term interest rates	Shiu (2004); Dorofti & Jakubik (2015)	Batool & Sahi (2019)	?
Penetration (gross prem to GDP)			?
Crisis (period)		Marović et al. (2010); Baluch et al. (2011)	-
Solvency II (period)		Jurkonytė & Girdzijauskas (2010)	-

## Empirical analysis

### Summary statistics & correlation analysis

The summary statistics of the mean values of the key variables at the firm level used in the model estimation are in Τable 3. The *firm size* ranged from 3.4 billion Euro to 854.7 billion Euro. The *capitalization ratio (total equity to total assets)* varied from 2.8% to 27.6%. The *use of reinsurance* spanned from -15.7% (concerns one insurance group which had both insurance and reinsurance activities) to 17.2%.The *invested assets to total assets ratio* varied from 39.8% to 91.5%. The *net premium to equity insurance leverage ratio* ranged from 61.1% to 493.4% (Table 3).

**Table 3:** Variable mean values at the insurance group level

Company _Id	ROA	ROE	Equity to Assets	Use of Reinsurance	Invested Assets to Total Assets	Net premium to Equity	Firm Size (Millions of Euros)
1	0.69%	8.10%	8.06%	4.78%	65.66%	244.90%	29,752.27
2	1.10%	10.47%	10.54%	9.78%	64.34%	186.21%	43,628.27
3	0.79%	8.22%	9.07%	3.52%	69.38%	91.50%	152,557.33
4	2.56%	34.51%	7.22%	4.38%	71.56%	289.78%	9,758.73
5	5.21%	24.59%	22.23%	5.95%	80.22%	168.09%	7,342.93
6	4.25%	15.58%	27.62%	4.46%	86.95%	61.09%	38,289.07
7	0.73%	9.92%	7.37%	0.52%	64.43%	166.73%	790,648.87
8	0.55%	13.38%	4.13%	0.48%	91.50%	206.84%	374,691.53
9	1.46%	10.37%	14.11%	1.36%	39.81%	204.34%	37,444.53
10	1.06%	15.71%	6.80%	8.29%	72.38%	126.25%	854,655.93
11	2.14%	18.83%	11.91%	12.13%	59.56%	207.48%	57,292.33
12	1.14%	10.96%	10.39%	3.88%	77.86%	193.36%	259,545.53
13	0.36%	12.62%	2.76%	4.28%	49.72%	493.36%	27,373.13
14	0.63%	14.51%	4.43%	3.53%	75.56%	337.44%	476,601.47
15	0.59%	8.37%	6.95%	6.43%	79.85%	272.99%	23,953.27
16	0.68%	9.30%	7.59%	2.92%	67.68%	238.37%	71,082.87
17	0.28%	4.29%	6.25%	-15.70%	42.32%	122.00%	380,284.80
18	4.63%	30.30%	16.54%	3.45%	84.08%	134.20%	37,921.87
19	2.27%	11.67%	19.13%	9.27%	64.20%	158.28%	3,372.26
20	3.00%	18.51%	16.62%	17.20%	58.95%	162.33%	12,335.40
21	2.65%	20.57%	12.98%	14.20%	63.86%	229.60%	58,461.47
22	0.39%	10.96%	3.73%	9.75%	64.00%	204.69%	442,431.60
23	1.43%	8.81%	18.02%	13.87%	58.12%	179.64%	24829.27
MeanAll	1.68%	14.37%	11.06%	5.60%	67.48%	203.46%	183,228.47
MaxAll	5.21%	34.51%	27.62%	17.20%	91.50%	493.36%	854,655.93
MinAll	0.28%	4.29%	2.76%	-15.70%	39.81%	61.09%	3,372.26

Source: Summary statistics of mean values at the firm level of the key variables used in estimation were obtained from the Thomson Reuters Datastream database (Refinitiv, 2022).

The summary statistics of the mean values of the key variables at the country level used in the model estimation are in Table 4. The *market size* ranged from 2.1 billion Euro in Slovenia to 256.8 billion Euro in the UK. The *market share held by the five largest insurers* spanned from approximately 40% in the UK to 82.4% in Finland. The *insurance penetration ratio* (ratio of gross (written) premium to GDP estimated per year and per country) ranged from 2.6% in Poland to 11.1 % in the Netherlands. The *gross domestic product annual growth* varied from -0.3% in Italy to 3.6% in Poland. The *inflation rate* ranged from 1.2% in France to 2.4% in Poland. the *long-term interest rates* spanned from 1.4% in Germany to 4% in Poland.

**Table 4:** Variable mean values at the country level

Country	Market Size (Million)	Five-Firm Concentration Ratio	GDP growth	Inflation Rate	Long Term Interest Rates	Penetration
Austria	17,010.29	72.08%	0.98%	1.91%	1.81%	5.08%
Belgium	27,805.34	63.17%	1.23%	1.87%	2.02%	6.94%
Denmark	26,861.15	60.31%	1.17%	1.39%	1.57%	9.87%
Finland	21,714.78	82.40%	0.77%	1.47%	1.71%	10.25%
France	207,274.16	47.19%	0.85%	1.21%	1.86%	9.63%
Germany	190,593.49	43.64%	1.06%	1.48%	1.41%	6.50%
Italy	148,118.55	58.63%	-0.30%	1.30%	3.17%	8.88%
Netherlands	76,642.83	65.88%	1.18%	1.69%	1.66%	11.08%
Poland	11,479.23	65.33%	3.61%	2.35%	4.01%	2.61%
Slovenia	2,070.76	78.10%	1.73%	1.62%	2.83%	5.17%
Spain	63,706.05	42.35%	0.37%	1.44%	2.86%	5.79%
United Kingdom	256,838.21	39.98%	0.98%	2.13%	2.33%	10.89%
Mean All	87,509.57	59.92%	1.14%	1.65%	2.27%	7.73%
Max All	256,838.21	82.40%	3.61%	2.35%	4.01%	11.08%
Min All	2,070.76	39.98%	-0.30%	1.21%	1.41%	2.61%

Source: Market size (measured by total premium) and the ratio of the market share held by the five largest insurers in each national market were obtained from Insurance Europe (2022). Annual growth in gross domestic product and inflation rate were sourced from the World Development Indicators (World Bank, 2022). Long-term interest rates come from the Federal Reserve Bank of St. Louis (2022) and the insurance penetration (ratio of total premium to GDP) was taken from the OECD (2022).

Table 5 reveals that the *ROA* has a positive correlation with each of *the ceded premiums/ gross premiums, investments/total assets, the five-firm concentration ratio, the annual growth of GDP* and *the long-term interest rates* and a negative correlation with *net premium/equity, the size of insurance group, the market size, the inflation rate* and *the penetration ratio*, as well as the *Crisis* and the *Solvency II* dummies. The *ROE* has similar correlations with *ROA* for each of the explanatory variables except for its correlation with *the five-firm concentration ratio,* which is negative. The *financial strength* of insurance groups (measured by the ratio of total equity (assets-liabilities) to total assets) has a positive correlation with each of the *ceded premium/gross premium, investments/total assets,* the *five-firm concentration ratio,* the *annual growth of GDP,* the *inflation rate,* and the *Solvency II* dummy and a negative correlation with *net premium/equity,* the *size of the insurance group,* the *market size,* the *long-term interest rates,* the *penetration ratio*, and the *Crisis* dummy. The strongest correlation (in absolute value) between independent variables is -0.7742 between the *five-firm consecration ratio* and *the market size*.

**Table 5:** Correlation coefficients for the dependent variables ROA, ROE, & Equity /Assets

Variables	ROA	ROE	Equity to Assets	Ceded Prem to Gross Prem	Investments to Assets	Net Prem to Equity	Size	Market Size	Five-Firm Concentration Ratio	GDP growth annual %	Inflation Rate	Interest Rates	Gross Written Prem to GDP	Crisis Dummy	Solvency II Dummy
ROA	1														
ROE		1													
Equity to Assets			1												
Ceded Prem to Gross Prem	0.1928	0.168	0.2559	1											
Investments to Assets	0.2818	0.27	0.1477	0.1013	1										
Net Prem to Equity	-0.3349	-0.0307	-0.5178	-0.0017	-0.19	1									
Size	-0.5	-0.2545	-0.5396	-0.3374	0.0403	-0.105	1								
Market Size	-0.4346	-0.2055	-0.4438	-0.0434	-0.1608	0.2538	0.6048	1							
Five-Firm Concentration Ratio	0.1951	-0.0074	0.294	-0.2744	0.1846	-0.246	-0.3253	-0.7742	1						
GDP growth annual %	0.1453	0.1411	0.0839	-0.016	0.0133	-0.0738	-0.0354	-0.104	0.0524	1					
Inflation Rate	-0.0137	-0.0709	0.0109	0.0011	-0.0541	0.054	-0.0626	-0.1094	0.0321	0.3068	1				
Long Term Interest Rates	0.0889	0.0453	-0.0016	0.0205	-0.046	0.2271	-0.1703	-0.1285	0.0112	-0.0705	0.3914	1			
Gross Written Prem to GDP	-0.1413	-0.1337	-0.0668	-0.301	-0.0615	-0.0124	0.2474	0.472	-0.1426	-0.0978	-0.1232	-0.0723	1		
Crisis Dummy	-0.0463	-0.1215	-0.0393	-0.0542	-0.1245	0.224	-0.0835	-0.0386	-0.0337	-0.2012	0.4132	0.6238	0.0153	1	
Solvency II Dummy	-0.0576	-0.0124	0.0121	0.0727	0.084	-0.2035	0.0954	0.0479	0.0479	0.0776	-0.1719	-0.7266	-0.0331	-0.5774	1

Source: Author’s calculations with data from the Datastream database (Refinitiv, 2022), Federal Reserve Bank of St. Louis (2022), Insurance Europe (2022), and the World Development Indicators (World Bank, 2022).

## Results and Discussion

Table 6 reveals the factors that impact profitability (captured by *ROA* and *ROE*) and *financial strength* (reflected by the equity-to-assets ratio). The regression results show that the *ratio of invested assets to total assets* and *GDP annual growth* posted a positive and statistically significant impact on both *profitability* metrics (0.1% and 1% levels, respectively) and *financial strength* (1% level). The former shows that insurers invested properly the excess of premium of earlier years and earnings of earlier investments minus their cost. The latter indicates that the insurance sector and the national economy somehow move together. When the economy grows, the insurers’ profitability and strength increase, as noted also by Apergis & Poufinas (2020).

**Table 6:** Regression Results

	ROA	ROE	Equity/ Assets
Ceeded Prem/Gross Prem	-0.0253**	-0.108	-0.0563**
	-0.024	-0.289	-0.05
Investments/Assets	0.0291****	0.153****	0.0300***
	0.0000	0.000	-0.006
NetPrem/Equity	-0.00621****	-0.0093	-0.0321****
	0.0000	-0.231	0.0000
Size	-0.00591****	-0.0126**	-0.0263****
	0.0000	-0.029	0.0000
Market Size	-0.00291**	-0.0322**	-0.00689
	-0.039	-0.022	-0.371
Five-Firm Concentration Ratio	-0.0412****	-0.359****	-0.0494
	0.0000	0.0000	-0.368
GDP growth annual %	0.0689***	0.397***	0.0795***
	-0.003	-0.008	-0.004
Inflation Rate	-0.157*	-1.476***	-0.0863
	-0.051	-0.007	-0.492
Long Term Interest Rates	0.115*	1.026*	-0.025
	-0.079	-0.066	-0.899
Gross Written Prem to GDP	0.0272	0.11	0.121
	-0.468	-0.791	-0.401
Crisis Dummy	-0.00061	-0.0310**	0.00438
	-0.746	-0.03	-0.18
Solvency II Dummy	-0.000689	0.00565	-0.000956
	-0.743	-0.745	-0.833
_cons	0.130****	0.761****	0.547****
Number of obs	345	345	345
R-squared	51.58	0.2262	0.4415
Chi-square	283.94	71.28	160.39
Prob>ch2	0.0000	0.0000	0.0000

Note: p-values in parentheses (* p<0.10, ** p<0.05, *** p<0.01, **** p<0.001)

Source: Author’s calculations with data from Datastream (Refnitiv, 2022), Federal Reserve Bank of St. Louis (2022), Insurance Europe (2022), and the World Development Indicators (World Bank, 2022).

In contrast, *(firm) size* exhibits a negative and statistically significant effect on both *profitability* metrics (at least at the 5% level) and *financial strength* (0.1% level). This negative correlation is potentially a consequence of the recent (non-organic growth) through mergers and acquisitions (M&A’s) observed in the insurance sector. Such growth takes several years to deliver results as a complete organizational restructuring requires time. This was subscribed to by Jakubik & Zafeiris (2018) who realized that, under the then current low-yield environment, insurers were changing their business models and looking for new investment and business opportunities. This led to an increasing number of mergers and acquisitions with the objective of achieving sufficient returns. However, the authors’ results do not confirm the positive impact of such M&A strategies on either the acquirers’ share prices or on the (accomplishment of abnormal) shareholder returns. Another reason could be the intense competition among the largest players in the market in which they operate. It could also be that smaller groups are more successfully managed compared to bigger groups and can thus achieve higher profitability/financial strength ratios.

Furthermore, *reinsurance* (measured by the *ceded premium to gross premium ratio*) and the *net premium-to-equity ratio* had a negative and significant impact on *ROA* (5% and 0.1% levels, respectively), but not on *ROE* and the *equity-to-assets ratio* (5% and 0.1% levels, respectively). The former is justified by the observation that reinsurance decreases (insolvency) risk, but at the same time reduces potential profitability. A possible interpretation for the latter comes from the realization that most of the firms in the dataset had a *net premium-to-equity ratio* of over 150% (Table 3) which is a sign of excessive underwriting that leads to higher underwriting losses and possible insolvencies.

*The market size* and *concentration in the insurance market* (measured by the *five-firm concentration ratio*) had a negative and significant impact on both *ROA* and *ROE* (5% and 0.1% levels, respectively), but not on the *equity-to-assets ratio*. To explain that, one may observe that large insurance markets potentially create opportunities for new entrants or the expansion of existing insurer activities. This observation, combined with the high concentration in such insurance markets, may lead to increased competition among insurers. Consequently, profitability may decline.

*Long-term interest rates* had a positive and marginally significant effect on both *ROA* and *ROE* (10% level). In contrast, the impact of *inflation* was negative and marginally significant on *ROA* (at the 10% level) and significant on *ROE* (1% level). None of them posted a statistically significant impact on *the equity-to-assets ratio*. The explanation of both effects may be comparable. For life insurers, especially with long-term guarantees, who tend to invest in long(er)-term fixed income instruments, the interest rate increase is beneficial, because liabilities tend to decrease more than assets, as they tend to have longer duration than assets (Poufinas, 2022). Furthermore, high interest rates make the achievement of promised returns easier. Hence, they increase the profitability of the insurer that results from the excess of the attained return over the promised return to the policyholders. For non-life insurers, who traditionally invest in short(er)-term fixed income assets, an increase in interest rates leads to higher returns that may exceed the level of claims (and other expenses). When it comes to *inflation*, higher inflation may translate to a higher level of claims and other expenses. This may reduce the profitability of the non-life insurers if they do not re-price in order to cover these incremental costs. Life insurers face lower (real) returns from fixed income investments against unchanged (nominal) promised returns, as inflation destroys value. Furthermore, inflation limits the disposable income of the investors and the insured and thus reduces both the level of the premium paid and the invested amount. Consequently, the profitability of life insurers deteriorates.

The European sovereign debt *crisis* affected *profitability* negatively and firm *financial strength* positively, but was not statistically significant, except for the case of *ROE,* in which the debt *crisis* exerted a negative and statistically significant impact (5% level). The explanation offered stems from the justification given for the impact of interest rates on profitability. As (primarily life) insurance companies invested during the crisis in long(er)-term income securities so as to capture the higher yields at the long end of the yield curve, they saw their asset portfolio value increase less than the value of their liabilities as a result of the (material) drop in interest rates that took place during the crisis. The increase in the value of the liabilities was larger relative to the assets because the coupons were reinvested at lower interest rates and at the same time constitute a small part of the assets. Moreover, liabilities tend to have higher duration than assets (duration gap). Hence their present value increased more than the present value of assets as a result of the interest rate decrease.

The impact of the implementation of *Solvency II* (*Solvency II* dummy) was positive for *ROE* and negative for *ROA* and *financial strength* but with no statistical significance in all cases. The lack of statistical significance may be attributed to the early (and gradual) preparation of large insurance groups in anticipation of the Solvency II requirements, years before it came into force. The approach introduced by Solvency II is financial in the sense that assets and liabilities are marked-to-market. As a result, there are differences in the valuation of assets and liabilities as per Solvency II compared to accounting purposes.^5^ This led insurance companies to increase their balance sheets for solvency purposes, regardless of the course of their income. In addition, it resulted in changes in investment policy, capital structure, volatility in the valuation of assets and liabilities, as well as in high implementation costs. The insurance groups in the present study are leaders in their country of origin and had both the know-how and the means to adopt the Solvency II Directive way in advance of its implementation.

### Effect of the pandemic

The years under investigation incorporated the years of the pandemic. The period was not examined separately as the consequences of the pandemic were still evolving at the time. COVID-19 had a notable impact during the period considered on individuals, society, business and the broader economic activity across the world and may, thus, be partly responsible for the results of this study. The insurance sector has not escaped its effects, but insurers responded quickly to this (newer) crisis. The pandemic brought challenges, but at the same time created new opportunities for insurers.

Life insurance activity might have been affected on a number of fronts as a result of the hit to economic activity and the employment levels at local, regional and global levels, which reduced consumer spending materially and swiftly, coupled with the market volatility and general uncertainty on consumer confidence and, therefore, willingness to spend. The most recent post-pandemic data are not yet available to permit discerning the final effect on premium levels. However, with respect to claims, despite the unprecedented number of lives lost due to the pandemic, which may continue to reverberate across society, insurers might not have seen a material increase in claims, as many of the diseased due to COVID-19 were older, belonging to age bands no longer covered by insurance; for example, in the European countries in which the insurance groups of our dataset are domiciled/are active, people older than 65 (or in some cases 67) years of age. For such individuals, life or (pure) endowment insurance policies expire at the age of 65 (or 67, respectively), since coverage is usually not offered above this age (as per the underwriting guidelines) or their insurance policies expired when they reached that age (according to their policy terms and conditions).

Focusing now on health insurance, we observe that the impact of the pandemic on health insurers will not be uniform as there is a wide diversity in health systems globally. Governments drove the treatment of infected patients and, therefore, the cost to health insurers might have been limited. Even though government spending on health care increased in light of the pandemic, the fear experienced by society may lead to higher penetration of private health insurance in the long term. The final effect of the pandemic on the *profitability* of health insurance remains to be seen in the years to come.

The impact on non-life insurance varied depending on the products and types of coverage offered. The pandemic had an impact on new sales (and thus premiums) in certain lines of business, such as travel, event, and trade credit insurance; losses from these lines of business were considerable. However, as countries reopen their boarders, travel, events and trade pick up again with a potential drop due to the continuation of remote presence, especially for professional purposes, established during the pandemic. Consequently, the corresponding insurance activities are anticipated to recuperate part of the losses realized during the COVID-19 years. Other lines of business, such as automobile insurance, were not affected as much. However, as people moved much less, claims volumes greatly decreased due to the lockdown(s). This might have led to re-pricing to lower premium levels in some cases. The net effect of these shifts of claims and premia on the *profitability* of non-life insurance should be unveiled within the next couple of years.

The aforementioned discussion indicates that the impact of the pandemic on the profitability of the insurance sector must be studied further as there are events that led to its decrease and events that led to its increase. Such a study can be performed when the 2022 data become available. However, a recent complication in Europe, starting at the end of 2021 – beginning of 2022, is the advent of the energy crisis as well as increasing inflation. This may complicate separation of the impact of the pandemic from that of the energy crisis and inflationary pressure.

## Conclusions

This study examined the factors that affect the profitability and financial strength of 23 insurance groups operating in the EU from 2007 to 2021. The findings reveal that the *efficiency of the management of accounts receivable*, *the state of the economy* and *long-term interest rates* had a positive and significant impact on the *profitability* of insurance groups; whereas, the *purchase of reinsurance*, *underwriting risk*, the *size of the insurance group*, *size of the domestic market*, *market structure* and the *inflation rate* had a negative and significant impact on the *profitability* of insurance groups. Furthermore, *the efficiency of the management of accounts receivable* and *state of the economy* had a significant and positive effect on the *financial strength* of insurance groups, whereas *underwriting risk* and the *size of the insurance group* had a significant and negative effect on the *financial strength* of insurance groups. Finally, the European sovereign debt *crisis* had a negative impact on the *profitability* (5% significance level on *ROE*) and a positive but insignificant impact on *financial strength*. Implementation of the *Solvency II* regulatory framework had a negative (positive) but insignificant impact on *ROA* (*ROE,* respectively) and a negative but insignificant impact on *financial strength*. These findings can provide valuable insight to insurers as well as to regulators regarding the determinants of the *profitability* and *financial strength* of insurance groups.

The research results indicate that insurance groups were able to withstand the crisis and perform all necessary changes well before the implementation of the Solvency II Directive. The latter means that they matched the capital required to perform the desired insurance activities with the risk emanating from these activities. As a matter of fact, this was the purpose of a series of studies/exercises lasting more than a decade that preceded implementation of the Solvency II Directive. Consequently, they were already in a position that was not affected by the actual implementation of the directive. This was the case for insurance groups. Smaller (local) insurers were not necessarily equally prepared. These findings indicate that adequate preparation to weather potential crises or upcoming regulatory changes may have a beneficial impact not only for the insurance industry, but also for the broader financial sector. This direction should be followed by competent authorities and policymakers; i.e. steer financial institutions to be better prepared for adverse conditions and upcoming legislation. Such an effort proved not to be in vain with the advent of the COVID-19 pandemic, as it found insurance groups (more than sufficiently) capitalized. The full impact of the pandemic on the profitability and financial strength of insurers will be captured when the latest data (including 2022) become available. Besides the challenges faced by the insurance sector during the pandemic, some new opportunities for increased insurance activity appear in the medium- to long-term horizon.

## Supplementary Information

ESM 1 (DOCX 17 kb)

## Footnotes

1 “The European sovereign debt crisis began in 2008 with the collapse of Iceland's banking system. Some of the contributing causes included the financial crisis of 2007 to 2008, and the Great Recession of 2008 through 2012. The crisis peaked between 2010 and 2012” (Kenton, 2021, p. 1 ).

2 “Solvency II is a Directive in EU law that codifies and harmonizes the EU insurance regulation. Primarily this concerns the amount of capital that EU insurance companies must hold to reduce the risk of insolvency” (Siddiqui, 2022, p. 1).

3 When calculating ROA and ROE, profits before taxes was ueed (as otherwise insurers from countries with higher tax rates would appear less profitable).

4 The reinsurance ratio is the ratio of the ceded premium to reinsurance over the total gross written premium. Ceded premium is the assigned portion of premiums collected by an insurance company to a reinsurer; at the same time part of the assumed risk is also transferred to the reinsurer.

5 For example, deferred acquisition costs and intangible assets are valued at zero in a Solvency II environment. Holdings in affiliated insurance and reinsurance undertakings are valued as stock market prices.
