# Supplementary material for: Impact of Internal and External Factors on the Profitability and Financial Strength of Insurance Groups
Source: Int Adv Econ Res. 2023 May 31:1–21. Online ahead of print. doi: 10.1007/s11294-023-09873-y (PMC10230474; doi:10.1007/s11294-023-09873-y)
Supplement: Supplementary file 1 — (DOCX 17 kb) [file 11294_2023_9873_MOESM1_ESM.docx]

**Online Supplemental Appendix**

**Table 1** EU Insurance Group selection for the dataset

| **Datastream Insurers (51)** | **Insurance Groups with data for the model variables (29)** | **Insurance Groups with data for the entire period (23)** | **Company_id** |
| --- | --- | --- | --- |
| Aegon NV | X | X | 1 |
| Ageas SA | X | X | 2 |
| Allianz SE | X | X | 3 |
| Allianz Slovenska Poistovnaas |  |  |  |
| Aon PLC |  |  |  |
| ASR Nederland NV | X |  |  |
| Assicurazioni Generali SpA | X | X | 4 |
| Assiteca SpA Internazionale di Brokeraggio Assicurativo |  |  |  |
| Atlantic Insurance Company Public Ltd |  |  |  |
| AXA SA | X | X | 5 |
| Aviva PLC | X | X | 6 |
| CNP Assurances SA | X | X | 7 |
| Coface SA | X |  |  |
| Cosmos Insurance PCL |  |  |  |
| DFV Deutsche Familienversicherung AG |  |  |  |
| European Reliance General Insurance Co SA |  |  |  |
| FBD Holdings PLC |  |  |  |
| Foyer SA | X |  |  |
| Grupo Catalana Occidente SA | X | X | 8 |
| Hannover Rueck SE | X | X | 9 |
| Interlife General Insurance Company SA |  |  |  |
| KD dd |  |  |  |
| KD Group dd | X |  |  |
| Life Star Holding plc |  |  |  |
| Linea DirectaAseguradora SA Compania de Seguros y Reaseguros |  |  |  |
| Mapfre Middlesea PLC |  |  |  |
| Mapfre SA | X | X | 10 |
| Minerva Insurance Company Public Ltd |  |  |  |
| Muenchener Rueckversicherungs Gesellschaft AG in Muenchen | X | X | 11 |
| Net Insurance SpA |  |  |  |
| NN Group NV | X |  |  |
| NUeRNBERGER Beteiligungs AG | X | X | 12 |
| Optimco NV |  |  |  |
| Pozavarovalnica Savadd |  |  |  |
| Regia Group dd |  |  |  |
| Powszechny Zaklad Ubezpieczen SA | X | X | 13 |
| Rhein Land Holding AG |  |  |  |
| RSA Insurance Group PLC | X | X | 14 |
| Sampo plc | X | X | 15 |
| Scor SE | X | X | 16 |
| Societa Cattolica di Assicurazione SpA | X | X | 17 |
| SynBiotic SE |  |  |  |
| Topdanmark A/S | X | X | 18 |
| Tryg A/S | X | X | 19 |
| Talanx AG | X |  |  |
| Unipol Gruppo SpA | X | X | 20 |
| Unipol Sai Assicurazioni SpA |  |  |  |
| UNIQA Insurance Group AG | X | X | 21 |
| Vienna Insurance Group AG Wiener Versicherung Gruppe | X | X | 22 |
| Wuestenrot & Wuerttembergische AG |  |  |  |
| Zavarovalnica Triglavdd | X | X | 23 |

Note: Out of 51 insurance groups and companies in the Datastream database (Refinitiv, 2022), 29 insurance groups had data for the variables of the models, of which 23 insurance groups had data for the entire period 2007-2021.
